# Comparative quantum-chemical investigation of 2-chloro-N-(4-methoxyphenyl)acetamide and 2-(4-methoxyphenylamino)-2-oxoethyl meth/acrylate: DFT, TD-DFT, and non-covalent interaction analyses

**DOI:** 10.1007/s10822-026-00871-w

**Published:** 2026-06-30

**Authors:** Nevin Çankaya, Mehmet Hanifi Kebiroğlu

**Affiliations:** 1 Vocational School of Health Services, Usak University, 64200 Uşak, Turkey; 2 Department of Medical Services and Techniques, Darende Bekir Ilicak Vocational School, Malatya Turgut Ozal University, 44700 Malatya, Turkey

**Keywords:** Density functional theory, TD-DFT, NBO analysis, Frontier molecular orbitals, Non-covalent interactions, Acrylate derivative

## Abstract

In this study, a comparative quantum-chemical investigation of 2-chloro-N-(4-methoxyphenyl)acetamide (p-acetamide), 2-(4-methoxyphenylamino)-2-oxoethyl acrylate (MPAEA), and 2-(4-methoxyphenylamino)-2-oxoethyl methacrylate (MPAEMA) was carried out to elucidate the effects of progressive structural modification on their electronic, spectroscopic, thermochemical, and non-covalent interaction properties. Geometry optimizations and electronic-structure calculations were performed within the framework of density functional theory using the 6-311G basis set. Electronic properties were analyzed through natural bond orbital (NBO) analysis, frontier molecular orbital (FMO) distributions, and global reactivity descriptors. The calculated HOMO–LUMO energy gaps revealed that MPAEA exhibits enhanced charge-transfer capability because of its conjugated acrylate structure, whereas MPAEMA shows a larger gap, suggesting higher electronic stability. Time-dependent density functional theory (TD-DFT) calculations were used to predict UV–Vis absorption features, revealing that structural modification significantly influences excitation energies and optical responses. Molecular electrostatic potential (MEP) maps and density of states (DOS/tDOS) analyses provided further insight into charge distribution and orbital contributions, highlighting increased electron delocalization in conjugated systems. Thermochemical analysis showed that thermal energy, heat capacity, and entropy increased systematically with temperature for all molecules, with MPAEMA exhibiting the highest thermodynamic values because of its extended molecular framework. Non-covalent interaction (NCI), density overlap regions indicator (DORI), and reduced density gradient (RDG) analyses revealed distinct weak-interaction patterns, confirming that structural complexity enhances interaction diversity and electron-density distribution. Overall, the results indicate that the transformation from the acetamide framework to acrylate and methacrylate derivatives significantly modifies the electronic structure, optical behavior, thermodynamic response, and interaction topology of methoxyphenyl-based molecular systems.

## Introduction

Aromatic amide derivatives represent an important class of organic compounds that have been widely studied in medicinal chemistry, materials science, and molecular electronics because of their structural versatility and tunable electronic properties [1]. Their physicochemical stability and functional-group diversity make them valuable scaffolds for pharmaceutical intermediates and advanced organic materials [2]. In addition, aromatic amide frameworks often participate in intermolecular interactions, such as hydrogen bonding and π–π stacking, which play critical roles in determining molecular stability, chemical reactivity, and spectroscopic behavior [3]. Methoxy-substituted phenyl derivatives have also attracted considerable interest because electron-donating substituents, such as the methoxy group, can substantially modulate the electron-density distribution of aromatic systems [4]. These electronic effects influence charge-transfer processes, orbital interactions, and molecular reactivity, thereby making methoxyphenyl frameworks suitable platforms for designing molecules with tailored electronic and optical properties [5]. When unsaturated acrylate or methacrylate fragments are incorporated into such aromatic amide scaffolds, additional π-conjugation, electron delocalization, and functional-group complexity can be introduced. However, the extent to which these structural modifications systematically alter electronic stability, optical response, thermochemical behavior, and weak-interaction topology remains insufficiently understood.

Modern computational chemistry methods, particularly density functional theory (DFT), have become indispensable tools for investigating the structural and electronic properties of molecular systems [8]. DFT-based approaches enable reliable analyses of molecular geometries, electronic structures, and spectroscopic responses, whereas complementary techniques, such as natural bond orbital (NBO) analysis, frontier molecular orbital (FMO) theory, and time-dependent DFT (TD-DFT) calculations, provide deeper insight into charge delocalization and electronic transitions [9]. In addition, molecular electrostatic potential (MEP) mapping and density-of-states (DOS) analysis allow detailed interpretation of charge distribution and orbital contributions [10]. The characterization of weak interactions using non-covalent interaction (NCI) analysis, reduced density gradient (RDG) visualization, and density overlap regions indicator (DORI) analysis has further improved the interpretation of subtle electronic effects that govern molecular stability and reactivity.

Previous theoretical studies have demonstrated that integrated DFT-based optoelectronic analyses are effective for explaining charge-transfer behavior and electronic responses in heteroaromatic and conjugated systems [11]. Similarly, combined computational approaches incorporating structural, thermochemical, and non-covalent interaction analyses provide a comprehensive framework for understanding structure–property relationships in complex organic molecules [12]. The combined use of HOMO–LUMO analysis, vibrational spectroscopy, and electrostatic potential mapping has also been shown to correlate electronic structure with physicochemical properties in functional molecular systems [13]. Previous investigations of acrylic acid derivatives have revealed that unsaturated conjugated systems show marked variations in UV–Vis, NMR, and vibrational features depending on their electronic configurations [14]. Studies of heteroaromatic carboxylic acid derivatives have also confirmed a strong relationship between structural descriptors and optoelectronic properties [15], whereas spectroscopic investigations of trimethoxy-substituted aromatic compounds have shown that methoxy substitution significantly modulates electron density and optical response [16]. In this context, methoxyphenyl-substituted oxoethyl methacrylate derivatives have been reported to display diverse spectroscopic and electronic behavior, highlighting their suitability for detailed quantum-chemical investigations [17]. Molecular modeling studies of methacrylate-based systems further emphasize the role of ester-linked conjugated fragments in determining electronic distribution, stability, and interaction profiles [18].

Despite these advances, the available literature still lacks a unified and comparative quantum-chemical assessment of how progressive structural transformation from a chloroacetamide framework to acrylate and methacrylate derivatives affects the electronic and interaction properties of a common methoxyphenyl-based scaffold. Most previous studies have focused either on individual molecules or on isolated properties, such as geometry, frontier orbital energies, vibrational features, or general molecular descriptors. Consequently, it remains unclear whether the introduction of an acrylate fragment enhances electron delocalization and optical activity more effectively than methacrylate substitution and how this structural progression influences thermochemical response and weak-interaction topology under identical computational conditions. This issue represents the specific knowledge gap addressed in the present work.

Therefore, this study presents a comparative quantum-chemical investigation of 2-chloro-N-(4-methoxyphenyl)acetamide (p-acetamide), 2-(4-methoxyphenylamino)-2-oxoethyl acrylate (MPAEA), and 2-(4-methoxyphenylamino)-2-oxoethyl methacrylate (MPAEMA). These three molecules were deliberately selected because they constitute a structurally related series built around a common methoxyphenyl-containing scaffold. p-acetamide represents the parent chloroacetamide framework, MPAEA introduces an acrylate fragment with extended π-conjugation, and MPAEMA incorporates an additional methyl-substituted methacrylate unit, thereby increasing molecular size, functional-group diversity, and structural complexity. This molecular design enables a direct evaluation of how stepwise structural modification controls electron delocalization, frontier-orbital distribution, TD-DFT excitation behavior, thermochemical properties, and weak-interaction patterns [19, 20].

The novelty of this work is not limited to the individual characterization of the selected compounds; rather, it lies in establishing a coherent structure property relationship across an acetamide acrylate methacrylate series using the same computational protocol. For this purpose, geometry optimization, NBO analysis, FMO evaluation, TD-DFT calculations, MEP mapping, DOS/tDOS analysis, thermochemical calculations, and NCI/RDG/DORI analyses were applied. The findings are expected to clarify the electronic consequences of increasing structural complexity and provide deeper insight into the design principles governing methoxyphenyl-based functional organic systems [8].

## Computational methods

All quantum-chemical calculations were carried out using Gaussian 09, Revision D.01, within the framework of density functional theory (DFT) [8]. Geometry optimizations of p-acetamide, MPAEA, and MPAEMA were performed without imposing symmetry constraints using the B3LYP hybrid exchange–correlation functional combined with the 6-311G basis set [21]. Harmonic vibrational frequency calculations were conducted at the same level of theory to verify that the optimized geometries corresponded to true minima on the potential energy surface, as confirmed by the absence of imaginary frequencies [22]. Natural bond orbital (NBO) analysis was performed to investigate intramolecular donor–acceptor interactions, second-order perturbation stabilization energies, and electron-delocalization features within the molecular frameworks [21]. Frontier molecular orbital (FMO) analysis was carried out to determine the energies and spatial distributions of the highest occupied molecular orbital (HOMO) and lowest unoccupied molecular orbital (LUMO) [23]. The HOMO–LUMO energy gap (ΔE) was used as a descriptor of electronic stability and chemical reactivity. Based on the frontier-orbital energies, conceptual DFT descriptors, including ionization potential (I), electron affinity (A), chemical hardness (η), softness (S), electronegativity (χ), chemical potential (μ), and electrophilicity index (ω), were calculated [24]. Excited-state properties and electronic absorption spectra were investigated using time-dependent density functional theory (TD-DFT) at the same theoretical level [25]. Solvent effects were not included in the TD-DFT calculations because the main objective of this work was to compare the intrinsic electronic and optical responses of the three structurally related molecules under identical theoretical conditions. Thus, the gas-phase approach provided a common reference framework for isolating the effects of structural modification on excitation energies, oscillator strengths, and dominant orbital transitions. The calculated UV–Vis data were therefore discussed in terms of relative electronic trends rather than direct solvent-dependent experimental absorption maxima. The calculated excitation energies, oscillator strengths, and dominant orbital transitions were analyzed to interpret the UV–Vis absorption features of the studied molecules [26].

Molecular electrostatic potential (MEP) surfaces were generated to visualize charge distribution and identify electron-rich and electron-deficient regions [27]. Density-of-states (DOS) and total density-of-states (tDOS) analyses were performed to evaluate the contributions of occupied and virtual molecular orbitals to the electronic structure [28]. Thermochemical parameters, including thermal energy (E), heat capacity at constant volume (C_v_), and entropy (S), were obtained from harmonic vibrational frequency calculations using standard statistical thermodynamic relations [29]. Weak non-covalent interactions were analyzed using non-covalent interaction (NCI), reduced density gradient (RDG), and density overlap regions indicator (DORI) approaches [30]. Molecular structures and graphical representations were prepared using GaussView 6 [31], whereas DOS, tDOS, NCI, RDG, and DORI analyses were generated using Multiwfn [32]. Recent comparative DFT and spectroscopic investigations have shown that systematic structural modification can significantly perturb frontier orbital distributions, electronic descriptors, and spectroscopic responses, thereby providing a useful framework for interpreting structure–property relationships in small organic molecular systems [33].

Previous DFT-based investigations on methacrylate-related systems have provided useful insight into electronic-structure features and structure property relationships [34]. Advanced computational investigations incorporating solvent effects, topological descriptors, and quantum-chemical parameters have also demonstrated the utility of integrated theoretical descriptors in interpreting functional organic molecules [35]. In addition, comprehensive analyses based on RDG-NCI frameworks have highlighted the critical role of non-covalent interactions in governing intermolecular behavior, stability, and reactivity in complex molecular systems [36]. To ensure methodological consistency, the Computational Methods section was restricted to the analyses actually performed in the present study. All post-processing analyses were limited to the computational descriptors directly reported in the present work. Accordingly, the methodological description was restricted to quantum-chemical, spectroscopic, thermochemical, and non-covalent interaction analyses.

Equations (1–9) quantitatively describe the electronic structure and chemical reactivity of the investigated molecules by defining chemical hardness (η), the inversely related softness (σ), electronegativity (χ), and chemical potential (μ). Electronegativity reflects the tendency of a molecule to attract electrons, whereas chemical potential characterizes the tendency of electrons to escape from the molecular system. These equations further define the electrophilicity index (ω), which indicates the electron-accepting ability of a molecule, the nucleophilicity index (ε), which reflects its electron-donating tendency, and the electron-accepting and electron-donating powers, expressed as ω^+^ and ω^−^, respectively. Collectively, these parameters, together with the HOMO–LUMO energy gap (ΔE), provide a quantitative framework for evaluating the kinetic stability and intrinsic chemical reactivity of the studied compounds. This focused description ensures internal coherence by aligning the theoretical definitions with the reported computational results.

1 $$ I = - E_{HOMO} $$

2 $$ A = - E_{LUMO} $$

3 $$ \eta = \frac{1}{2}\left[ {\frac{{\partial^{2} E}}{{\partial^{2} N}}} \right]_{v(r)} = \frac{I - A}{2} $$

4 $$ \left\langle \alpha \right\rangle = \frac{1}{3}\left[ {\alpha_{xx} + \alpha_{yy} + \alpha_{zz} } \right] = \sigma = \frac{1}{\eta } $$

5 $$ \mu = - \chi = \left[ {\frac{\partial E}{{\partial N}}} \right]_{V(r)} = - \left( {\frac{I + A}{2}} \right) $$

6 $$ \omega = \frac{{\chi^{2} }}{2\eta } $$

7 $$ \varepsilon = \frac{1}{\omega } $$

8 $$ \omega^{ + } = \frac{{\left( {I + 3A} \right)^{2} }}{{16\left( {I - A} \right)}} $$

9 $$ \omega^{ - } = \frac{{\left( {3I + A} \right)^{2} }}{{16\left( {I - A} \right)}} $$

### Synthesis of molecules

**Fig. 1:**
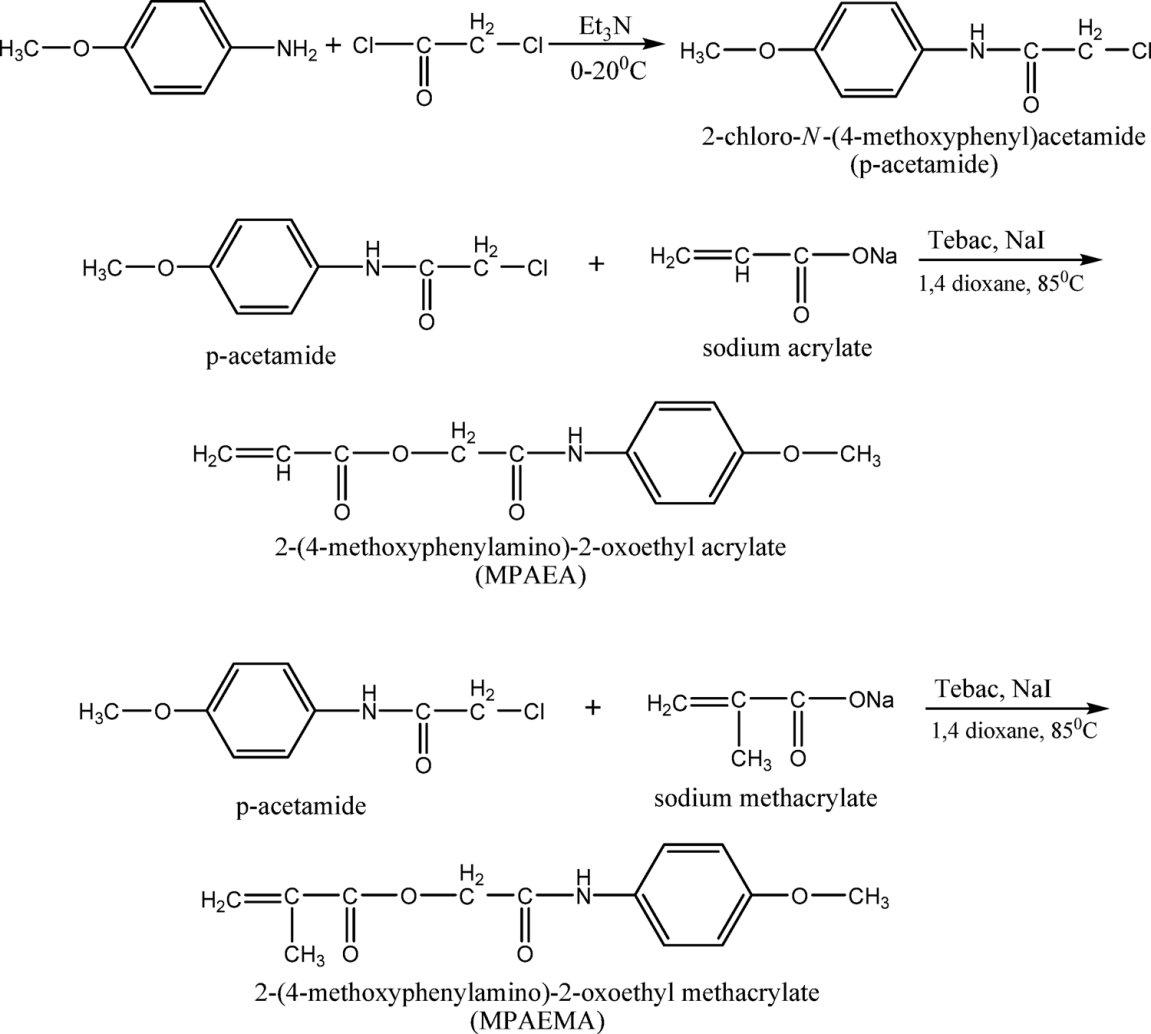
Reaction scheme for the synthesis of p-acetamide, MPAEA, and MPAEMA

The investigated molecules, namely 2-chloro-N-(4-methoxyphenyl)acetamide (p-acetamide), 2-(4-methoxyphenylamino)-2-oxoethyl acrylate (MPAEA), and 2-(4-methoxyphenylamino)-2-oxoethyl methacrylate (MPAEMA), were previously synthesized and characterized by our research group [34]. The synthetic route for these molecules is presented in Fig. 1.

## Results and discussion

### Natural bond orbital (NBO) analysis

**Fig. 2:**
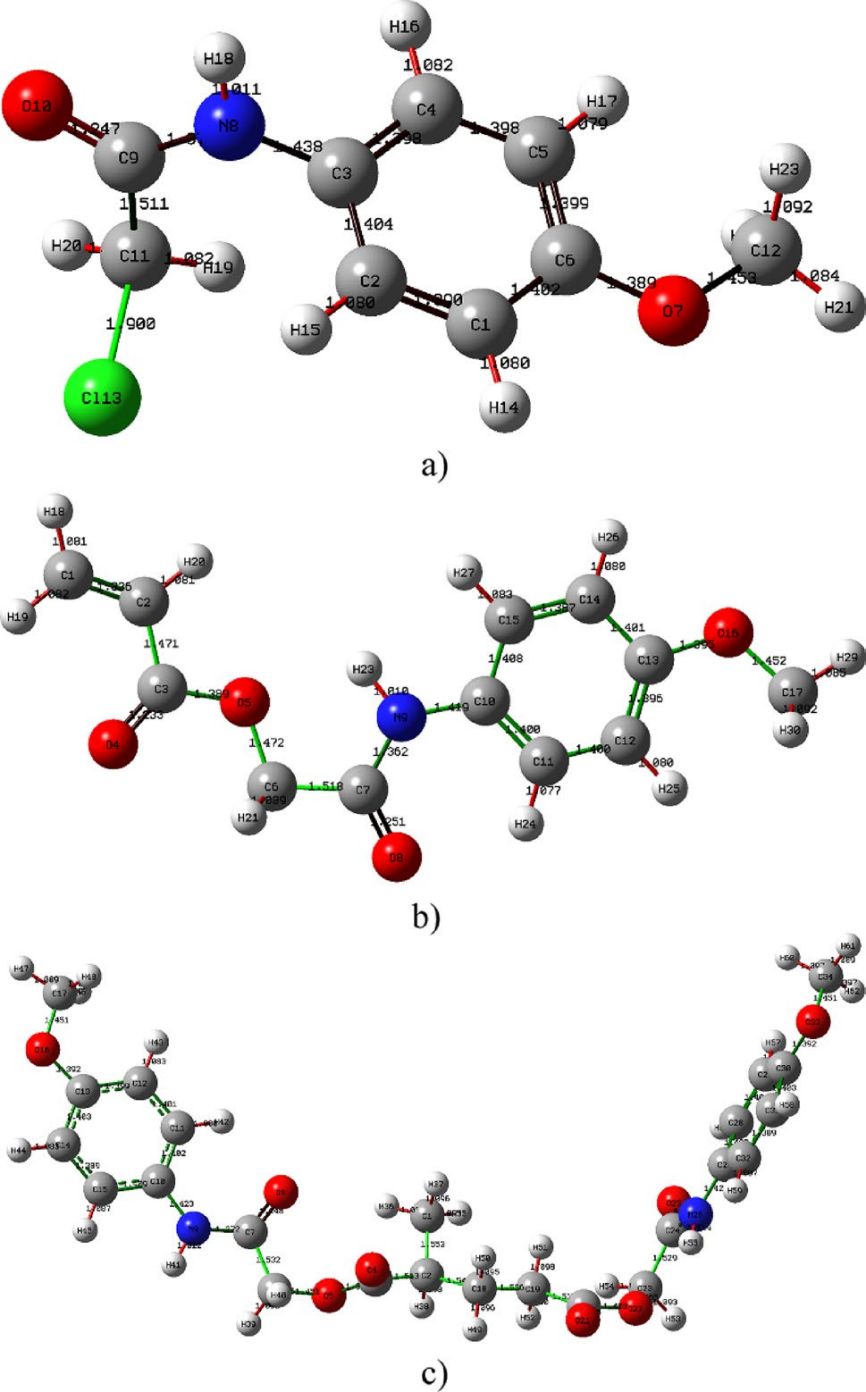
NBO of **a** p-acetamide, **b** MPAEA, **c** MPAEMA

The major donor–acceptor interactions are summarized in Table 1, and the corresponding orbital-interaction schemes are presented in Fig. 2. For the p-acetamide molecule, stabilization primarily arises from π-electron delocalization within the aromatic ring and lone-pair interactions involving heteroatoms. Lone-pair electrons on the carbonyl oxygen contribute to n(O) → π*(C=O) interactions, thereby supporting resonance stabilization of the amide functionality. In addition, the nitrogen lone pair interacts with adjacent antibonding orbitals, enhancing conjugation between the aromatic ring and the amide fragment. In the MPAEA molecule, the NBO results reveal stronger donor acceptor interactions than those observed for p-acetamide. This enhancement is mainly attributed to the conjugated acrylate fragment, which extends the π-electron system and promotes more efficient electron delocalization. As illustrated in Fig. 2, the π orbitals of the aromatic ring interact with the antibonding π* orbitals of the acrylate group, leading to pronounced π → π* charge-transfer interactions. Lone-pair electrons on oxygen atoms participate in n(O) → π*(C=O) interactions associated with the ester functionality, thereby increasing stabilization energies. For the MPAEMA molecule, distinct electronic behavior is observed. Although the molecule contains extended functional groups, the NBO analysis indicates that donor–acceptor interactions are comparatively less effective in promoting long-range delocalization than those in MPAEA. This behavior can be attributed to the methacrylate structure, in which additional substituents introduce partial electronic localization and reduce conjugation efficiency across the molecular backbone. Although π → π* interactions remain present, they are less delocalized, whereas n(O) → π*(C =O) interactions are more localized around the ester groups rather than contributing to extended charge transfer. The stabilization energies, E(2), indicate that MPAEA exhibits the most effective intramolecular charge-transfer character among the studied systems, followed by MPAEMA and p-acetamide. This trend reflects the role of conjugation continuity and functional-group arrangement in governing electron delocalization. The results show that acrylate incorporation enhances global electron delocalization, whereas methacrylate substitution introduces structural complexity that partially limits conjugation, leading to a more localized but electronically stable framework. These findings are consistent with the subsequent frontier molecular orbital (FMO) analysis, in which variations in HOMO–LUMO energy gaps further reflect differences in electronic reactivity.

**Table 1:** Selected second-order perturbation stabilization energies, E(2), obtained from NBO analysis of p-acetamide, MPAEA, and MPAEMA

Molecule	Donor NBO	Acceptor NBO	E(2) (kcal mol^−1^)	Assignment
p-acetamide	LP(1) N8	BD*(2) C9-O10	66.48	Amide N → carbonyl π* resonance
	LP(2) O7	BD*(2) C5-C6	29.44	Methoxy O → aromatic π* delocalization
	LP(2) O10	BD*(1) N8-C9	22.72	Carbonyl O → amide antibonding interaction
	BD(2) C1-C2	BD*(2) C5-C6	22.71	Aromatic π → π* delocalization
	BD(2) C3-C4	BD*(2) C1-C2	21.39	Aromatic π → π* delocalization
MPAEA	LP(1) N9	BD*(2) C7-O8	74.35	Strong amide-type resonance
	LP(2) O5	BD*(2) C3-O4	42.29	Lone-pair-assisted carbonyl delocalization
	LP(2) O4	BD*(1) C3-O5	35.47	Oxygen lone-pair donation
	LP(1) N9	BD*(2) C10-C11	32.47	N lone-pair contribution to π-system
	LP(2) O16	BD*(2) C12-C13	27.59	Acrylate-related O → π* interaction
MPAEMA	LP(1) N9	BD*(2) C7-O8	68.39	Amide N → carbonyl π* resonance
	LP(1) N26	BD*(2) C24-O25	62.79	Amide N → carbonyl π* resonance in the second unit
	LP(2) O5	BD*(2) C3-O4	42.62	Oxygen lone-pair-to-carbonyl π* delocalization
	LP(2) O22	BD*(2) C20-O21	39.32	Oxygen lone-pair-to-carbonyl π* delocalization
	LP(2) O21	BD*(1) C20-O22	36.97	Localized oxygen lone-pair stabilization
	LP(2) O4	BD*(1) C3-O5	36.16	Oxygen lone-pair-assisted stabilization
	LP(1) N26	BD*(2) C27-C28	32.31	Nitrogen lone-pair contribution to π-delocalization
	LP(1) N9	BD*(2) C10-C11	30.95	Nitrogen lone-pair contribution to π-delocalization

The selected second-order perturbation stabilization energies, E(2), obtained from NBO analysis are summarized in Table 1. For p-acetamide, the dominant interaction was LP(1) N8 → BD*(2) C9-O10, with an E(2) value of 66.48 kcal mol^−1^, suggesting strong amide resonance stabilization. MPAEA exhibited a higher stabilization energy for the corresponding LP(1) N9 → BD*(2) C7-O8 interaction, with an E(2) value of 74.35 kcal mol^−1^, supporting its enhanced donor acceptor delocalization character. Additional strong interactions in MPAEA, including LP(2) O5 → BD*(2) C3–O4 and LP(1) N9 → BD*(2) C10-C11, further indicate that the acrylate-containing framework promotes extended charge delocalization. For MPAEMA, the strongest interactions were associated with LP(1) N9 → BD*(2) C7-O8 and LP(1) N26 → BD*(2) C24-O25, with E(2) values of 68.39 and 62.79 kcal mol^−1^, respectively. These values suggest that MPAEMA possesses multiple localized resonance-stabilization pathways rather than a single extended delocalization route.

### Frontier molecular orbital (FMO) analysis

**Fig. 3:**
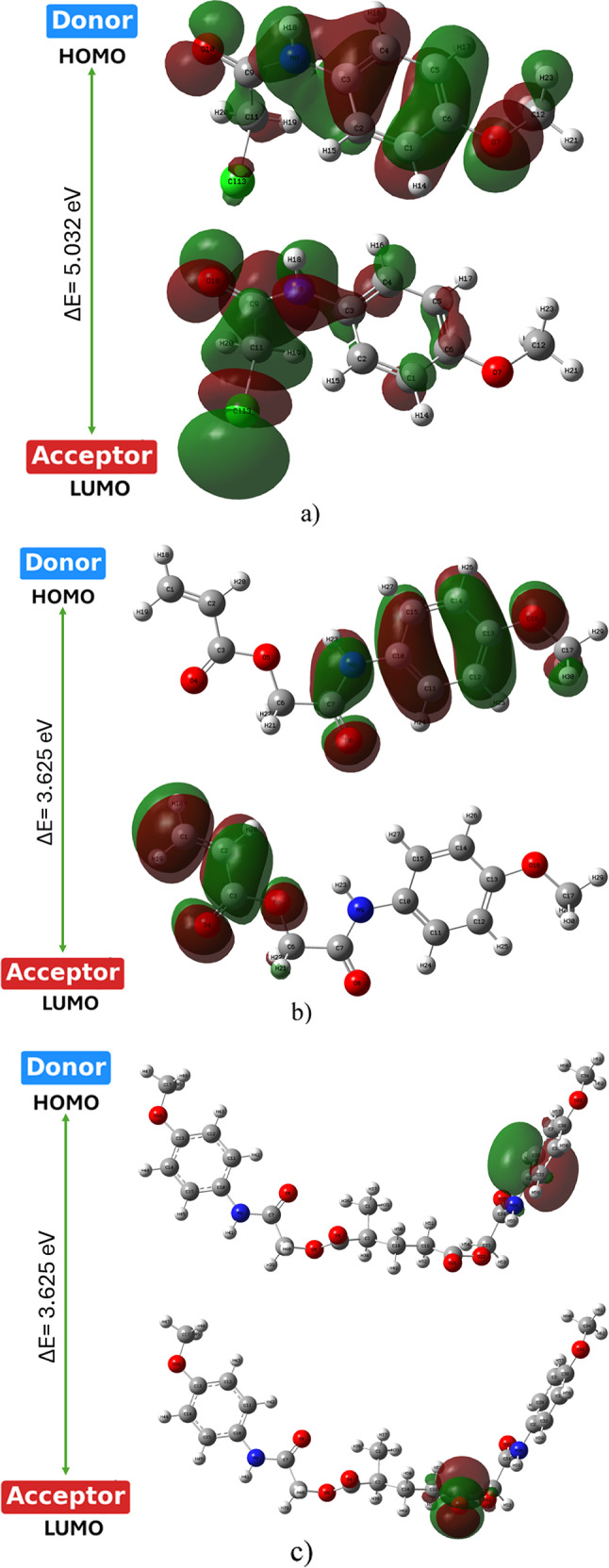
Frontier molecular orbital distributions and energy level diagrams of **a** p-acetamide, **b** MPAEA, and **c** MPAEMA. For meaningful comparison

The orbital distributions are presented in Fig. 3, and the calculated frontier-orbital energies and global reactivity descriptors are summarized in Table 2. For the p-acetamide molecule, the HOMO and LUMO energies were calculated as − 6.599 and − 1.567 eV, respectively, resulting in an energy gap of 5.032 eV. This relatively moderate gap reflects balanced electronic stability and limited charge-transfer capability. The HOMO was primarily localized over the aromatic ring and partially on the amide group, whereas the LUMO was mainly concentrated around the carbonyl region and aromatic π-system. In the MPAEA molecule, the HOMO and LUMO energies were − 5.808 and − 2.183 eV, respectively, giving a significantly reduced energy gap of 3.625 eV. This decrease in ΔE indicates enhanced electronic polarizability and increased chemical reactivity. The charge-transfer tendency of MPAEA should therefore be interpreted in a comparative descriptor-based context. Among the studied molecules, MPAEA exhibited the smallest HOMO–LUMO energy gap, suggesting higher electronic polarizability and easier intramolecular electronic redistribution. This result is consistent with its higher softness value and bathochromically shifted TD-DFT transition relative to those of p-acetamide and MPAEMA. In addition, the NBO results showed relatively strong donor acceptor stabilization interactions in MPAEA, supporting more effective electron delocalization through the conjugated acrylate fragment. However, because charge-transfer distance and transition-density analyses were not explicitly performed, MPAEA is described as having the most pronounced charge-transfer tendency rather than an unequivocally measured charge-transfer character. The HOMO was more delocalized over both the aromatic ring and the conjugated acrylate fragment, whereas the LUMO was largely localized on the electron-deficient acrylate and carbonyl groups. This spatial separation facilitates intramolecular charge transfer, consistent with the strong donor–acceptor interactions observed in the NBO analysis.

**Table 2:** The calculated quantum chemical descriptors of a) p-acetamide b) MPAEA c) MPAEMA

Parameter	Values		
	p-acetamide	MPAEA	MPAEMA
*E*_HOMO_ (eV)	− 6.599	− 5.808	− 6.838
*E*_LUMO_ (eV)	− 1.567	− 2.183	− 0.768
Δ*E* (eV)	5.032	3.625	5.620
η (eV)	2.516	1.813	2.810
σ (eV^−1^)	0.397	0.552	0.356
χ (eV)	4.083	3.996	3.578
μ (eV)	− 4.083	− 3.996	− 3.578
ω	3.313	4.404	2.277
ɛ	0.302	0.227	0.439
ω^+^	5.37	4.45	0.84
ω^−^	1.29	1.60	4.42

For the MPAEMA molecule, the HOMO and LUMO energies were calculated as − 6.838 and − 0.768 eV, respectively, resulting in the largest energy gap of 5.620 eV among the studied systems. This increased ΔE suggests higher electronic stability and reduced charge-transfer capability compared with those of both p-acetamide and MPAEA. The HOMO was relatively localized and primarily distributed over the aromatic region, whereas the LUMO was shifted toward the ester and methacrylate-related moieties. However, unlike MPAEA, the conjugation pathway in MPAEMA appeared less continuous, limiting long-range electron delocalization and reducing electronic reactivity. The global reactivity descriptors further support this interpretation. The chemical hardness (η) increased from 1.813 eV for MPAEA to 2.810 eV for MPAEMA, whereas the softness (σ) decreased accordingly, confirming the more rigid and less reactive electronic structure of MPAEMA. MPAEA exhibited the highest softness and electrophilicity index (ω = 4.404), suggesting a stronger tendency to accept electrons and participate in charge-transfer processes. The electrophilicity index of MPAEMA (ω = 2.277) was significantly lower, reflecting reduced electrophilic character and enhanced stability. The practical implications of the softness and electrophilicity descriptors provide further insight into the molecular behavior of the investigated systems. Softness reflects the ease with which the electron density of a molecule can be polarized or redistributed during interactions with external electrophilic or nucleophilic species. Thus, a higher softness value indicates greater electronic flexibility and higher chemical responsiveness. In this context, MPAEA, which exhibited the highest softness value among the studied molecules, is expected to undergo electronic redistribution more easily than p-acetamide and MPAEMA. The electrophilicity index, on the other hand, describes the tendency of a molecule to accept electronic charge. The higher electrophilicity value of MPAEA suggests a stronger electron-accepting tendency and supports its more pronounced charge-transfer tendency. In contrast, the lower softness and electrophilicity values of MPAEMA indicate a more electronically rigid and comparatively less reactive molecular framework, consistent with its larger HOMO–LUMO energy gap and higher chemical hardness. Therefore, MPAEA emerges as the most electronically reactive system, whereas MPAEMA represents a more stable but less delocalized molecular framework. These findings are consistent with the NBO results and coherently explain the structure–reactivity relationship in methoxyphenyl-based molecular systems.

To contextualize the FMO results, a comparison was made with earlier studies on methacrylate derivatives. Earlier work on MPAEMA at the B3LYP/6-311 +  + G(d,p) level showed that HOMO–LUMO energies are sensitive to molecular structure and conjugation [34]. The HOMO and LUMO distributions were further interpreted in relation to molecular reactivity. The HOMO regions indicate the electron-donating parts of the molecules, whereas the LUMO regions represent the electron-accepting sites. Thus, orbital delocalization and the spatial relationship between the HOMO and LUMO provide insight into possible charge-redistribution pathways. MPAEA exhibited a more favorable orbital arrangement for electronic redistribution, consistent with its smaller HOMO–LUMO gap and higher softness. In contrast, the more localized orbital distribution of MPAEMA agrees with its larger energy gap and higher electronic stability. Overall, the present results confirm that the energy gap is a key indicator of electronic stability and reactivity. Minor differences may arise from the use of different basis sets. Both studies indicate that the methacrylate derivative exhibits a larger energy gap than the acrylate derivative, reflecting higher stability and reduced charge-transfer capability. For visual consistency and meaningful comparison, the HOMO and LUMO isosurfaces of all three molecules were generated using the same isovalue and graphical scaling parameters.

### UV–Vis spectroscopy

**Fig. 4:**
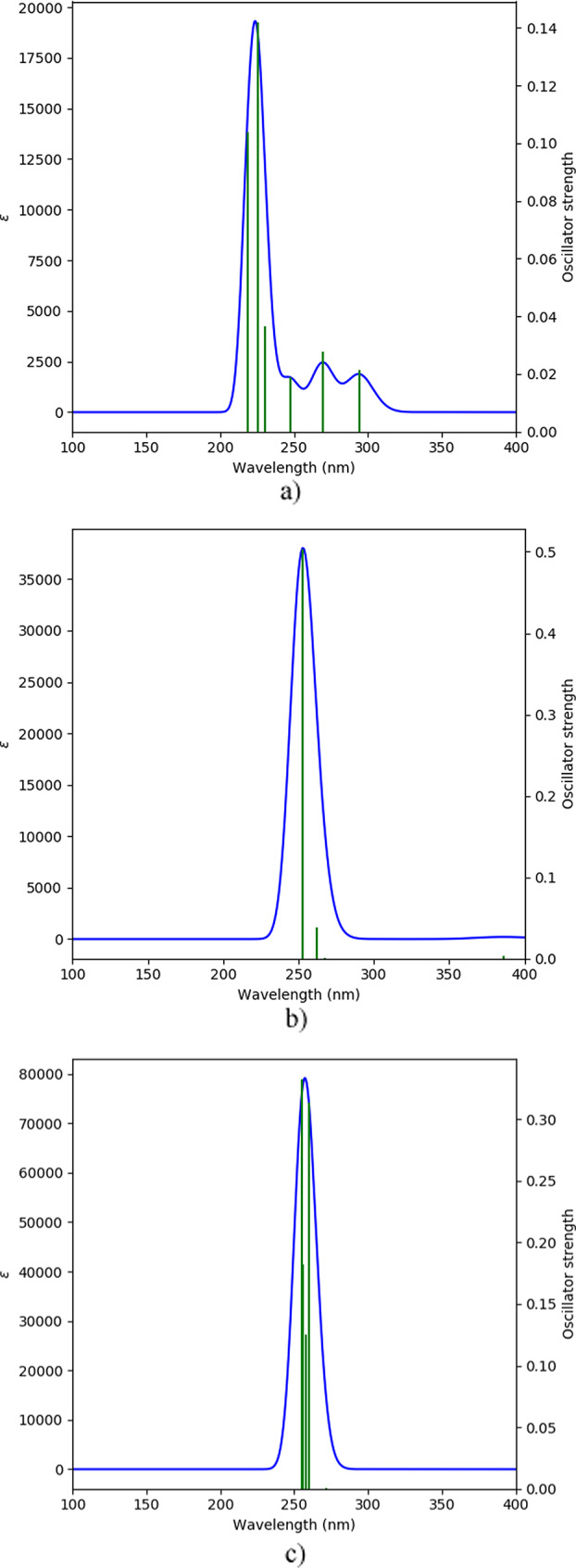
Simulated UV–Vis absorption spectra of **a** p-acetamide, **b** MPAEA, and **c** MPAEMA

The simulated UV–Vis spectra are presented in Fig. 4, whereas the excitation energies, oscillator strengths, and dominant orbital transitions are summarized in Tables 3, 4 and 5. For the p-acetamide molecule, the first singlet excitation (S1) appeared at 294.19 nm with an oscillator strength of 0.0213, mainly arising from HOMO → LUMO (55%) and H-1 → LUMO (41%) transitions. This transition is typical of a π → π* excitation localized within the aromatic ring and the adjacent carbonyl group. Additional transitions observed at shorter wavelengths, ranging from 269 to 247 nm, correspond to higher-energy excitations involving deeper occupied orbitals, reflecting a relatively localized electronic structure. The MPAEA molecule exhibited a pronounced bathochromic shift, with the first excitation appearing at 386.12 nm and being dominated entirely by the HOMO → LUMO transition (100%). This red shift is a direct consequence of the extended π-conjugation introduced by the acrylate fragment, which lowers the excitation energy and facilitates long-range intramolecular charge transfer. A strong absorption band at 252.58 nm with a high oscillator strength (f = 0.5035) was mainly attributed to the HOMO → L + 1 transition, suggesting multiple accessible excited states and enhanced optical activity. For MPAEMA, the first singlet excitation appeared at 271.91 nm with a low oscillator strength of f = 0.0004 and was mainly associated with the HOMO → L + 1 transition. Therefore, this transition should be considered a weak electronic excitation rather than an intense absorption band. The more pronounced transitions were observed for S2, S5, and S6, with oscillator strengths of 0.3138, 0.1820, and 0.3327, respectively. These transitions mainly involved HOMO → L + 2, HOMO → L + 5, and H − 1 → LUMO/H-1 → L + 4 contributions. The TD-DFT results indicate that MPAEMA does not show a strong low-energy S1 absorption; instead, its absorption intensity is mainly concentrated in higher-energy UV transitions. This behavior indicates that the electronic structure of MPAEMA is less favorable for low-energy charge-transfer excitations, which can be attributed to reduced conjugation continuity within the methacrylate framework. Comparative analysis of the three systems revealed that structural modification strongly influences optical properties. The MPAEA molecule displayed the most pronounced red shift and the strongest charge-transfer tendency, whereas p-acetamide showed intermediate behavior. MPAEMA was characterized by blue-shifted absorption features and higher excitation energies, reflecting a more localized electronic structure and reduced delocalization efficiency. The TD-DFT results show that incorporation of the acrylate group enhances optical activity and promotes low-energy electronic transitions, whereas methacrylate substitution introduces structural effects that shift absorption toward higher energies. These findings are consistent with the FMO analysis, in which variations in HOMO–LUMO energy gaps directly influence the observed optical behavior. The present TD-DFT calculations were performed in the gas phase. Therefore, the calculated absorption wavelengths should not be interpreted as direct solvent-corrected experimental λmax values. Instead, they provide a consistent theoretical basis for comparing the relative optical responses of p-acetamide, MPAEA, and MPAEMA. Within this framework, the bathochromic shift observed for MPAEA and the blue-shifted behavior of MPAEMA are interpreted as consequences of structural modification and differences in conjugation continuity rather than solvent-mediated stabilization effects.

**Table 3:** Calculated electronic excitations from TD-DFT analysis of p-acetamide

State	Energy (cm^−1^)	Wavelength (nm)	Oscillator strength (f)	Major electronic transition	Contribution (%)
S1	33,991	294.19	0.0213	HOMO → LUMO	55
				H-1 → LUMO	41
S2	37,122	269.38	0.0279	H-1 → LUMO	51
				HOMO → LUMO	43
S3	40,444	247.25	0.0188	HOMO → L + 1	79
				H-3 → L + 2	7
S4	43,411	230.36	0.0365	H-2 → LUMO	76
				H-4 → LUMO	8
S5	44,391	225.27	0.142	H-3 → LUMO	59
				HOMO → L + 2	25
S6	45,666	218.98	0.1037	HOMO → L + 2	54
				H-3 → LUMO	23

**Table 4:** Calculated electronic excitations from TD-DFT analysis of MPAEA

State	Energy (cm^−1^)	Wavelength (nm)	Oscillator strength (f)	Major electronic transition	Contribution (%)
S1	25,898	386.12	0.0029	HOMO → LUMO	100
S2	35,739	279.80	0.0001	H-4 → LUMO	31
				H-2 → LUMO	68
S3	37,403	267.35	0.0009	H-1 → LUMO	99
S4	37,751	264.88	0.0001	H-4 → LUMO	67
				H-2 → LUMO	31
S5	38,186	261.87	0.0382	H-1 → L + 1	11
				HOMO → L + 2	77
S6	39,591	252.58	0.5035	HOMO → L + 1	88

**Table 5:** Calculated electronic excitations from TD-DFT analysis of MPAEMA

State	Energy (cm^−1^)	Wavelength (nm)	Oscillator strength (f)	Major electronic transition	Contribution (%)
S1	36,776.46	271.9131	0.0004	HOMO → L + 1	94
S2	38,456.52	260.034	0.3138	HOMO → L + 2	71
				HOMO → L + 5	17
S3	38,750.91	258.0585	0.1256	H-1 → LUMO	31
				H-1 → L + 4	54
S4	38,825.92	257.5599	0.0001	HOMO → LUMO	99
S5	39,034.81	256.1816	0.1820	H-2 → L + 2	11
				HOMO → L + 2	17
				HOMO → L + 5	61
S6	39,223.55	254.9489	0.3327	H-1 → LUMO	61
				H-1 → L + 4	22

The electronic transitions were further interpreted according to their orbital character. In p-acetamide, the low-energy transitions mainly involved HOMO/H-1 → LUMO excitations localized over the aromatic ring and the carbonyl-containing amide region. Thus, these transitions can mainly be assigned to π → π* excitations, with possible n → π* contributions arising from heteroatom lone pairs associated with the carbonyl and methoxy groups. In MPAEA, the first excitation was dominated by the HOMO → LUMO transition and appeared at a longer wavelength than those of p-acetamide and MPAEMA. This transition can be assigned to a π → π* excitation with an intramolecular charge-transfer contribution because the conjugated acrylate fragment extends the electronic-delocalization pathway and facilitates electron redistribution from the methoxyphenyl region toward the electron-deficient acrylate/carbonyl moiety. In MPAEMA, the TD-DFT results indicate that the S1 transition has a low oscillator strength and should be considered a weak excitation. The more intense absorptions were mainly associated with higher-energy transitions involving HOMO → L + 2, HOMO → L + 5, and H-1 → LUMO/H-1 → L + 4 contributions. These transitions are best interpreted as higher-energy π → π* transitions with localized n → π* contributions from oxygen- and nitrogen-containing functional groups rather than as strong low-energy charge-transfer absorptions.

### MEP and DOS analysis

**Fig. 5:**
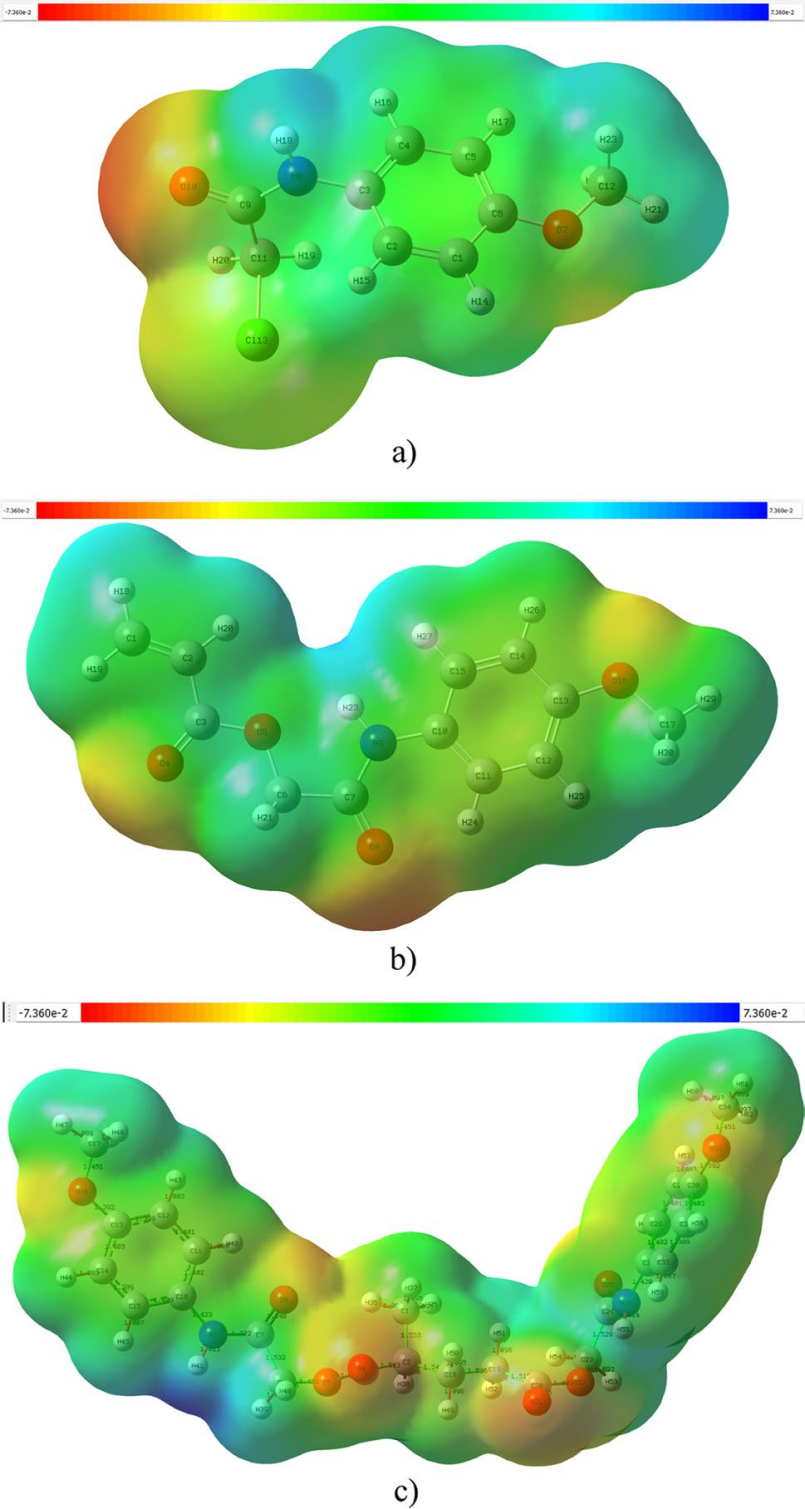
Molecular Electrostatic Potential of **a** p-acetamide, **b** MPAEA, **c** MPAEMA

The calculated MEP surfaces are presented in Fig. 5, where the electrostatic potential was mapped onto the electron-density surface using a color scale ranging from red, representing electron-rich regions, to blue, representing electron-deficient regions. For the p-acetamide molecule, the most negative electrostatic-potential regions were mainly localized around the carbonyl oxygen atom and partially around the methoxy oxygen atom, suggesting electron-rich sites that may act as nucleophilic centers. Positive-potential regions were primarily concentrated near the amide hydrogen atoms, suggesting electrophilic regions that can participate in hydrogen-bonding interactions. The aromatic ring showed intermediate potential values because of π-electron delocalization. In the MPAEA molecule, a broader and more extended negative electrostatic-potential distribution was observed, particularly around the ester carbonyl groups and the conjugated acrylate fragment. This distribution reflects enhanced electron delocalization across the molecular framework, leading to an expanded nucleophilic surface. The presence of the conjugated system promotes charge transfer from the methoxyphenyl donor region toward the electron-deficient acrylate moiety, consistent with the strong donor–acceptor interactions identified in the NBO and FMO analyses. For the MPAEMA molecule, the MEP surface revealed a more heterogeneous charge distribution. Although electron-rich regions were still localized around oxygen-containing functional groups, the negative potential was less continuously distributed across the molecular framework compared with that of MPAEA. This suggests a reduction in long-range electron delocalization and a more localized electronic structure. At the same time, multiple electron-rich centers were observed, suggesting that MPAEMA has several potential reactive sites rather than a single dominant charge-transfer pathway.

**Fig. 6:**
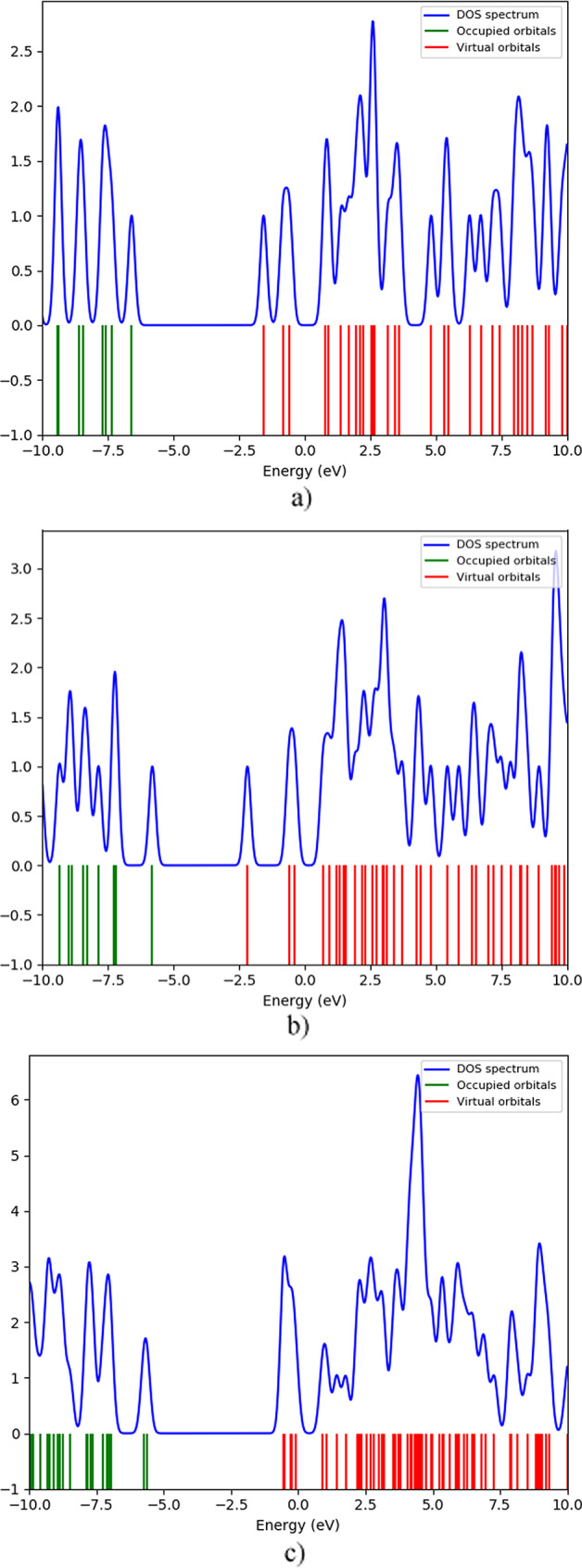
Density of States (DOS) of **a** p-acetamide, **b** MPAEA, **c** MPAEMA

**Fig. 7:**
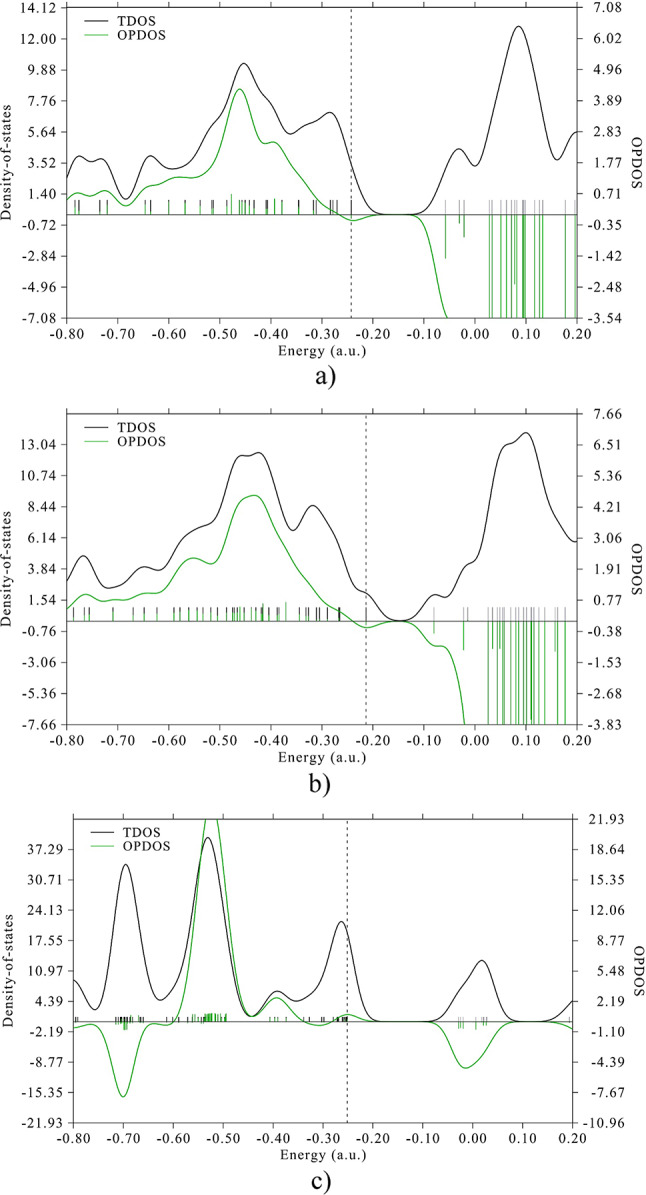
Total Density of States (tDOS) of **a** p-acetamide, **b** MPAEA, **c** MPAEMA

To further explain the electronic structure, DOS and total density-of-states (tDOS) analyses were performed, as shown in Figs. 6 and 7. For p-acetamide, a clear separation between occupied and unoccupied molecular orbitals was observed, consistent with its relatively large HOMO–LUMO energy gap. The MPAEA molecule showed a more compact distribution of electronic states near the frontier-orbital region, suggesting stronger orbital interactions and enhanced electron delocalization. This dense distribution facilitates electronic transitions and supports its observed red-shifted optical behavior. The MPAEMA molecule showed a comparatively wider separation between occupied and virtual states, particularly near the frontier region. This distribution reflects reduced orbital overlap and a lower density of states around the HOMO–LUMO region, which is consistent with its larger energy gap and blue-shifted absorption features. The tDOS profile further indicates that the electronic states in MPAEMA are more discretely distributed, supporting a more localized electronic framework. The combined MEP and DOS analyses show that structural transformation significantly influences charge distribution and orbital organization. Whereas MPAEA showed enhanced electron delocalization and a continuous charge-transfer pathway, MPAEMA was characterized by a more localized and structurally complex electronic environment. These findings agree with the NBO, FMO, and TD-DFT results and collectively confirm that conjugation continuity plays a critical role in determining the electronic and optical properties of methoxyphenyl-based molecular systems.

### Thermochemistry surface maps (TCSM) analysis

**Fig. 8:**
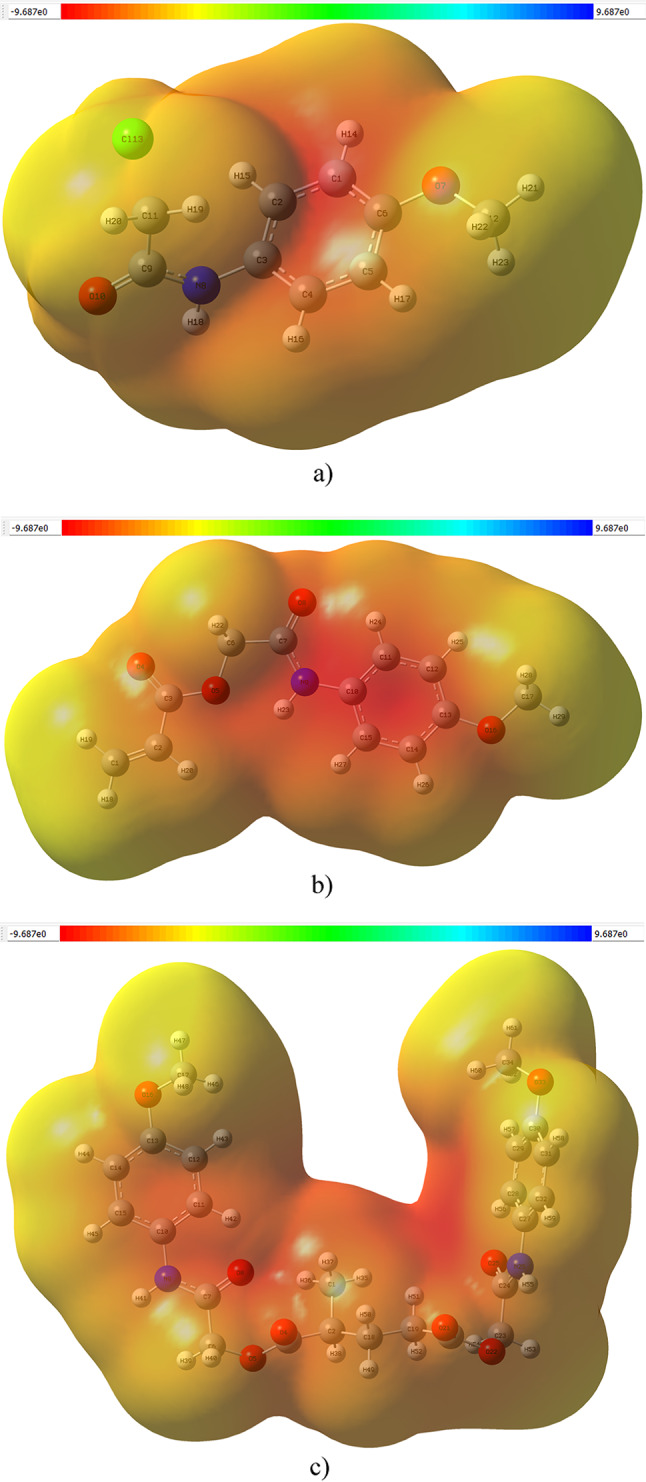
Thermochemistry surface maps of **a** p-acetamide, **b** MPAEA, and **c** MPAEMA generated from optimized geometries and harmonic vibrational frequency calculations

**Fig. 9:**
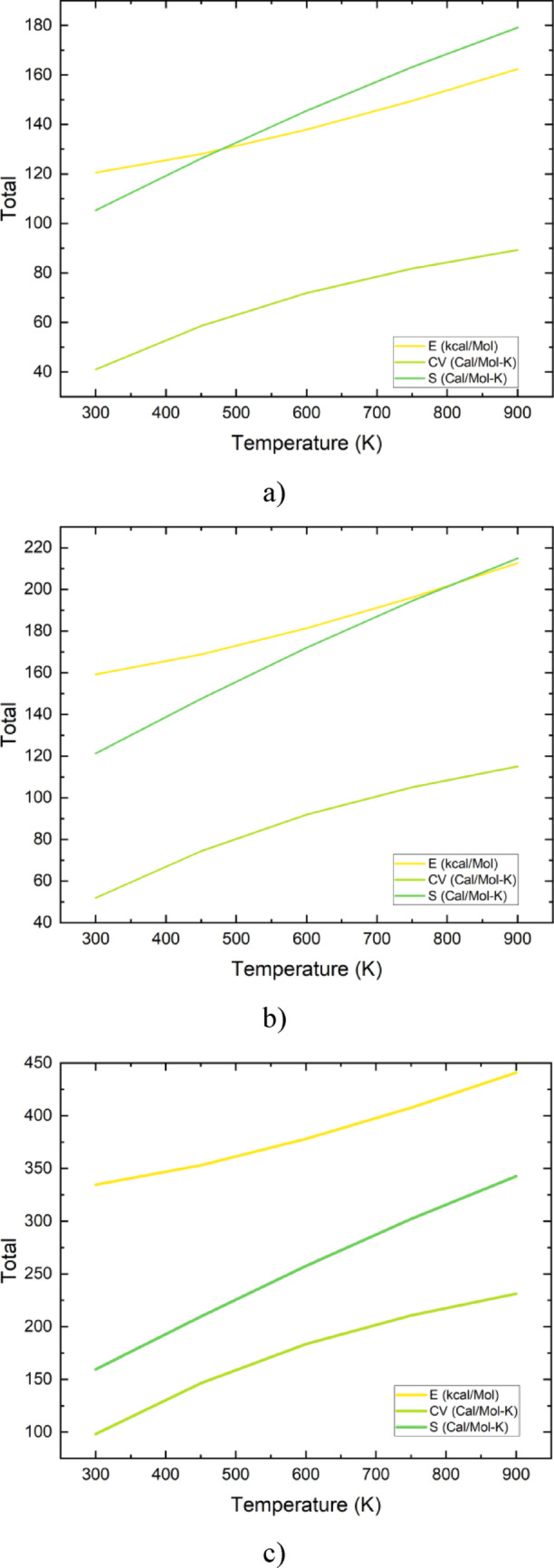
Total of **a** p-acetamide, **b** MPAEA, **c** MPAEMA

The thermochemical behavior of p-acetamide, MPAEA, and MPAEMA was evaluated using thermochemistry surface maps (TCSM) combined with temperature-dependent thermodynamic parameters. The thermochemistry surface maps shown in Fig. 8 were generated from the optimized molecular geometries and harmonic vibrational frequency calculations obtained at the B3LYP/6-311G level. The thermochemical quantities were evaluated using standard statistical thermodynamic relations derived from the vibrational frequency calculations. The color scale in the thermal surface maps represents the relative distribution of thermal/vibrational contributions over the molecular framework. Warmer colors indicate regions with relatively higher thermal contributions and stronger vibrational participation, whereas cooler colors correspond to regions with lower thermal contributions. Therefore, these maps should be interpreted as comparative visual representations of the spatial distribution of thermal response rather than as absolute temperature maps. The thermal surface distributions are presented in Fig. 8, whereas the variations in thermal energy (E), heat capacity at constant volume (C_v_), and entropy (S) with temperature are shown in Fig. 9 and summarized in Table 5. As illustrated in Fig. 8, the thermal surfaces revealed non-uniform energy distributions across the molecular frameworks. In p-acetamide, the thermal density was primarily localized around the aromatic amide region, suggesting a limited vibrational contribution from the structure. The MPAEA molecule showed a broader distribution extending over the conjugated acrylate fragment, suggesting increased participation of the extended π-system in thermal motion. The most pronounced behavior was observed for the MPAEMA molecule, in which the thermal surface was widely distributed across the entire molecular framework. This extensive distribution reflects the larger molecular size and increased structural complexity, which contribute to a greater number of accessible vibrational modes and enhanced energy dispersion.

The numerical data in Table 5 show that thermal energy, heat capacity, and entropy increased systematically with temperature for all molecules. For p-acetamide, the thermal energy increased from 120.565 to 162.340 kcal mol^−1^ between 300 and 900 K. In comparison, MPAEA showed higher values, ranging from 159.256 to 212.690 kcal mol^−1^. MPAEMA showed significantly higher thermodynamic values, with thermal energy increasing from 334.658 to 440.874 kcal mol^−1^ over the same temperature range. A similar trend was observed for heat capacity and entropy, with MPAEMA consistently displaying the highest values across all temperatures. This behavior can be attributed to the increased molecular mass, structural flexibility, and additional functional groups of MPAEMA, which collectively enhance its vibrational degrees of freedom. According to statistical thermodynamics, a larger number of accessible microstates leads to higher entropy and heat capacity, thereby explaining the observed trend. The extended framework of MPAEMA allows more efficient energy storage and redistribution under thermal excitation (Table6).

**Table 6:** Total of a) p-acetamide b) MPAEA c) MPAEMA

T(K)	E (kcal/Mol)			CV (Cal/Mol-K)			S (Cal/Mol-K)		
	p-acetamide	MPAEA	MPAEMA	p-acetamide	MPAEA	MPAEMA	p-acetamide	MPAEA	MPAEMA
300	120.565	159.256	334.658	41.066	51.899	98.241	105.324	121.246	159.482
450	128.082	168.782	353.125	58.554	74.422	146.438	126.208	147.492	209.543
600	137.919	181.325	378.011	71.924	91.962	183.456	145.554	171.999	257.587
750	149.484	196.148	407.669	81.795	105.059	210.640	163.160	194.439	302.038
900	162.340	212.690	440.874	89.293	115.065	231.210	179.128	214.879	342.704

### NCI, DORI, and RDG analysis

**Fig. 10:**
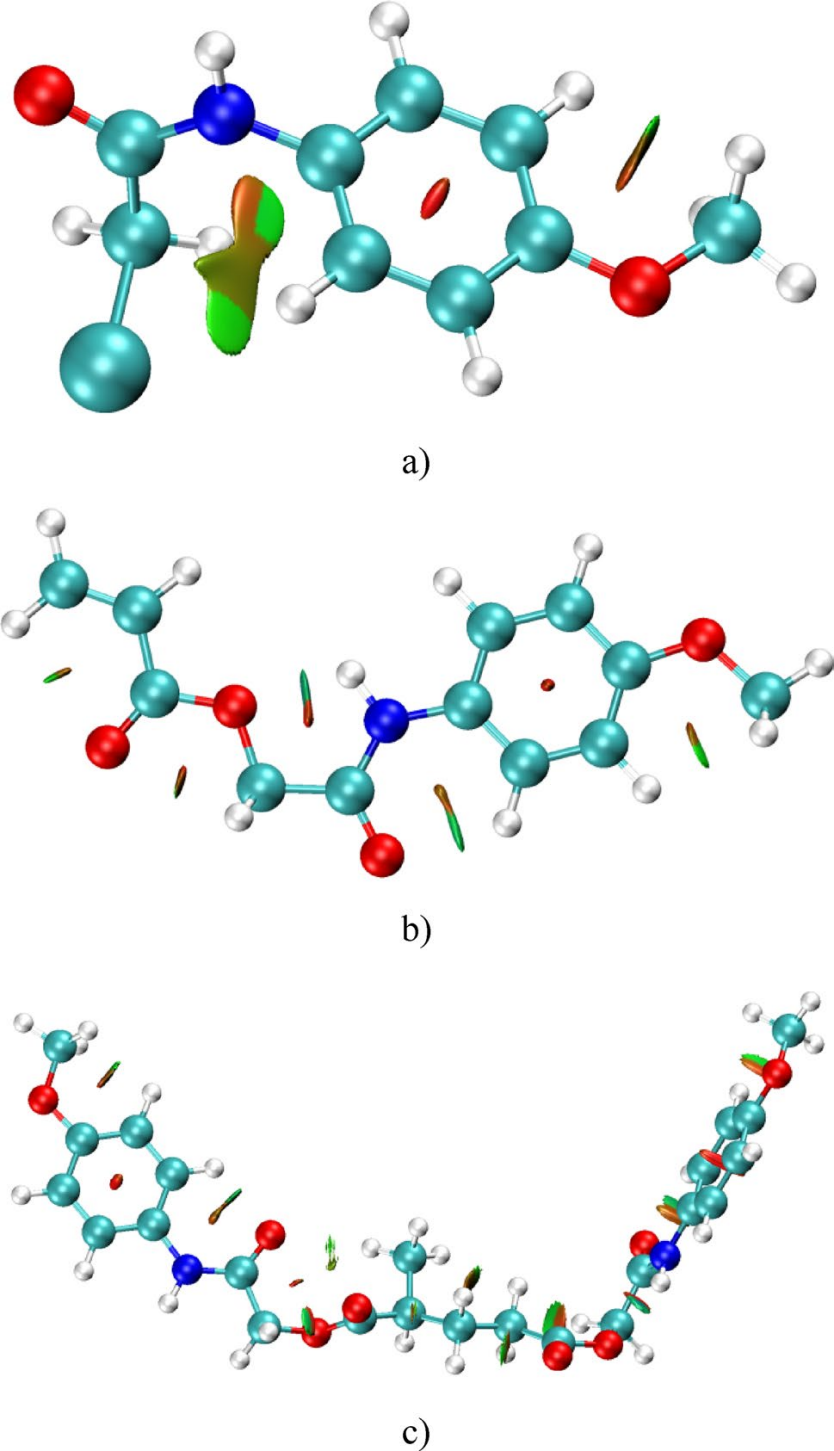
NCI of **a** p-acetamide, **b** MPAEA, **c** MPAEMA

**Fig. 11:**
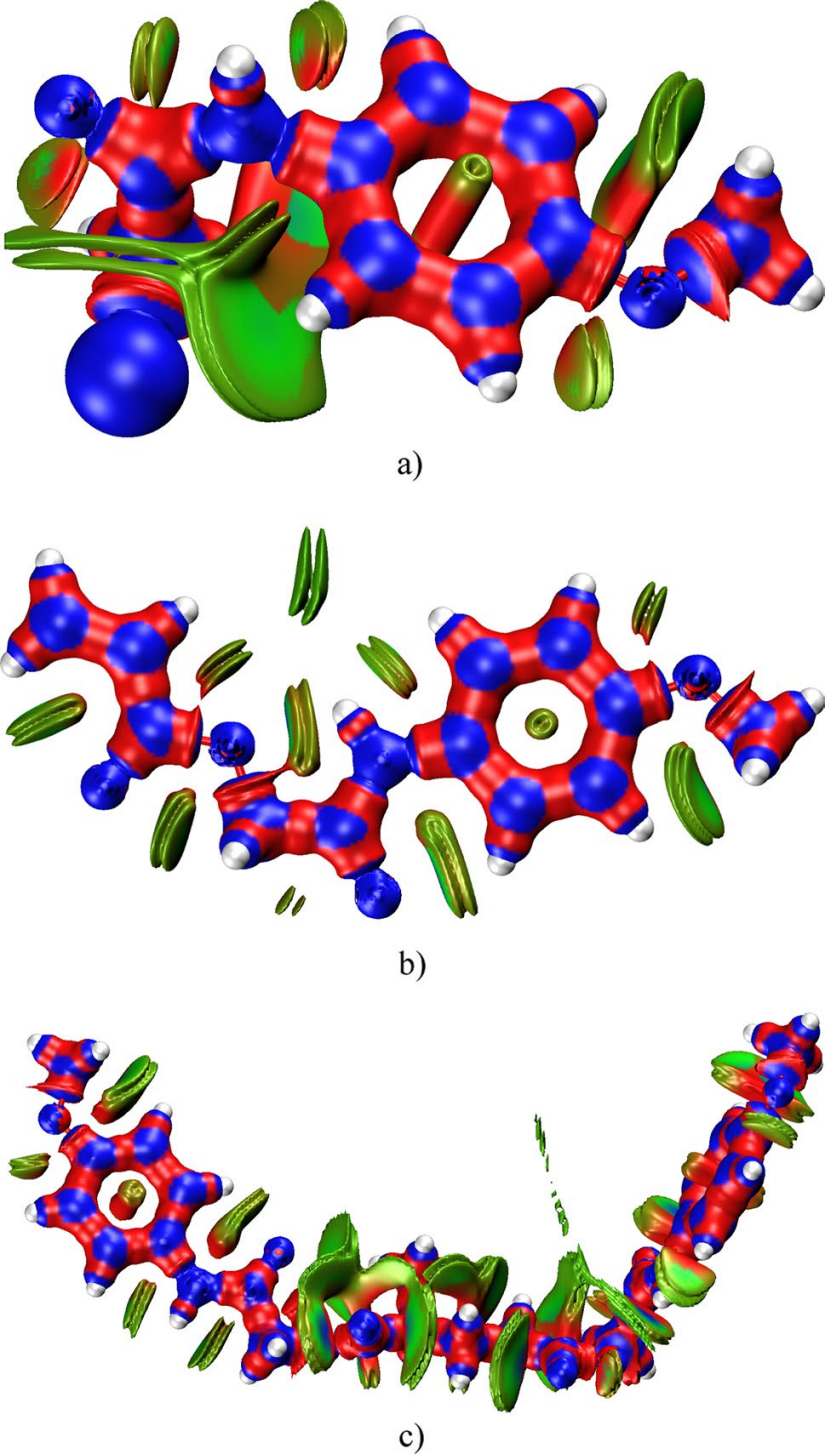
DORI of **a** p-acetamide, **b** MPAEA, **c** MPAEMA

**Fig. 12:**
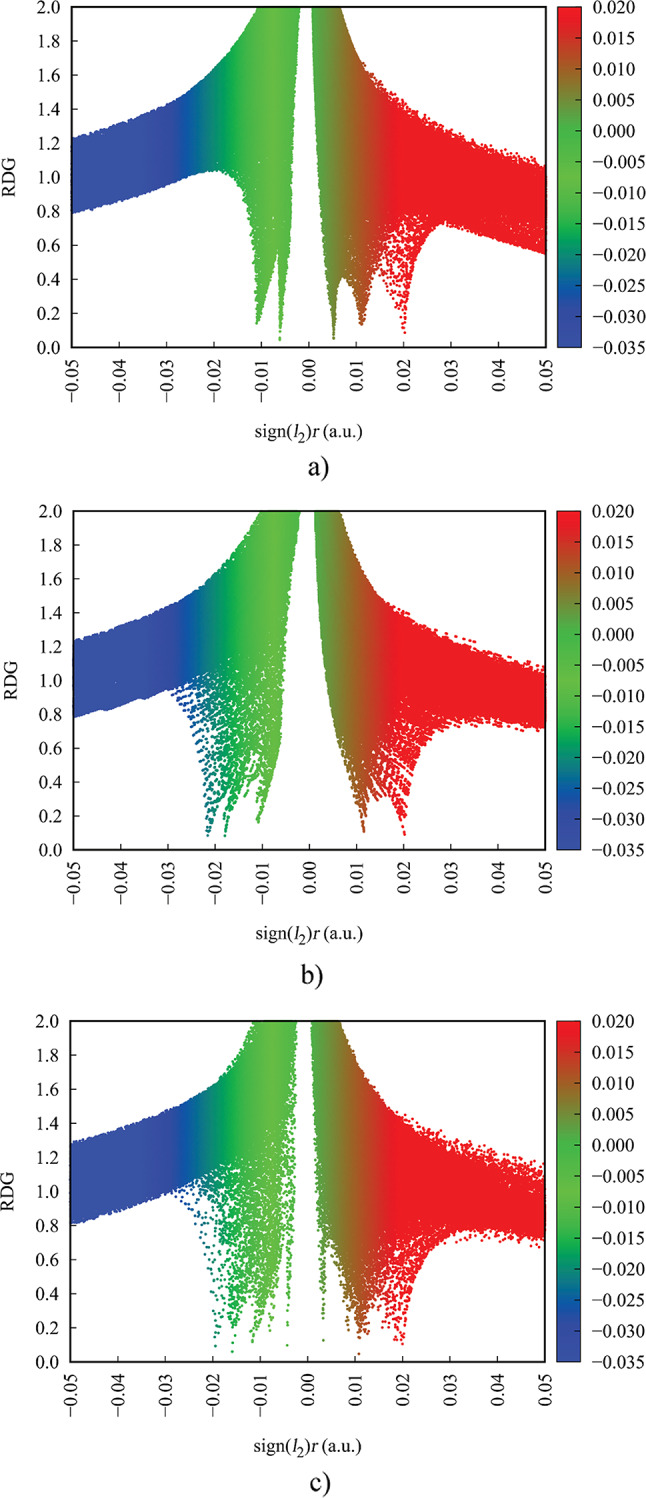
RDG of **a** p-acetamide, **b** MPAEA, **c** MPAEMA

The NCI isosurfaces, DORI maps, and RDG scatter plots of p-acetamide, MPAEA, and MPAEMA are presented in Figs. 10, 11 and 12, respectively. These analyses were used to evaluate the weak-interaction topology, electron-density overlap, and reduced-density-gradient behavior of the investigated molecules. Unlike MPAEA, in which interactions are largely guided by a continuous conjugated pathway, MPAEMA exhibited a more fragmented but spatially extensive interaction network. This suggests that structural complexity promotes the formation of multiple localized interaction domains rather than a single dominant interaction channel. The DORI maps provide further insight into electron-density overlap. In p-acetamide, electron density was primarily localized within the aromatic ring and amide group. For MPAEA, the DORI surfaces extended over both the aromatic and acrylate fragments, suggesting that delocalization increased across the conjugated system. The MPAEMA molecule showed multiple distinct regions of electron-density overlap, reflecting a heterogeneous distribution of electronic interactions throughout the molecular framework. The RDG scatter plots support these observations. For p-acetamide, the distribution of points was relatively narrow, suggesting limited weak-interaction diversity. The MPAEA molecule showed a broader distribution near the low-density-gradient region, characteristic of increased van der Waals and dispersive interactions. The MPAEMA system showed the widest distribution, with multiple regions corresponding to both attractive and repulsive interactions, suggesting a richer and more complex interaction landscape. The combined NCI, DORI, and RDG analyses show that structural modification significantly influences weak-interaction patterns. Whereas MPAEA enhanced interaction delocalization through conjugation, MPAEMA introduced structural complexity that increased interaction diversity and spatial distribution. These findings confirm that increasing molecular size and functional-group variety leads to a more complex interaction topology, which plays a critical role in determining molecular stability and physicochemical behavior.

To complement the interpretation of the NCI, DORI, and RDG analyses, the interaction regions were evaluated not only from the visual isosurfaces but also from the typical sign(λ_2_)ρ and RDG distribution patterns. In NCI analysis, negative sign(λ_2_)ρ values are associated with attractive interactions, near-zero values indicate weak van der Waals-type interactions, and positive values correspond to steric or repulsive contributions. p-acetamide mainly showed localized weak-interaction regions, whereas MPAEA showed a more extended distribution of weak attractive and dispersive interactions because of its conjugated acrylate framework. MPAEMA displayed a more heterogeneous interaction pattern, with multiple localized weak-interaction regions arising from its larger and more structurally complex methacrylate framework. These results should be interpreted as comparative evidence for differences in weak-interaction topology rather than as direct quantitative measures of binding strength. Therefore, the NCI/DORI/RDG results should be interpreted as comparative evidence for differences in weak-interaction topology rather than as direct quantitative measures of binding strength.

## Conclusion

In this study, a comprehensive quantum-chemical investigation was carried out to evaluate the structural, electronic, spectroscopic, thermochemical, and intermolecular-interaction properties of 2-chloro-N-(4-methoxyphenyl)acetamide (p-acetamide), 2-(4-methoxyphenylamino)-2-oxoethyl acrylate (MPAEA), and 2-(4-methoxyphenylamino)-2-oxoethyl methacrylate (MPAEMA) using density functional theory-based computational approaches. Geometry optimization confirmed the structural stability of all molecules and provided reliable configurations for subsequent analyses. Natural bond orbital (NBO) analysis revealed that incorporation of the acrylate fragment significantly enhanced donor acceptor interactions and intramolecular charge-transfer tendency, with MPAEA exhibiting the most effective electron delocalization. Frontier molecular orbital (FMO) analysis further supported these findings, showing that MPAEA exhibited the smallest HOMO–LUMO energy gap and the highest electronic reactivity, whereas MPAEMA exhibited the largest gap, suggesting greater electronic stability and reduced charge-transfer capability.

Time-dependent DFT calculations showed that structural modification strongly influences optical properties. The MPAEA molecule displayed a pronounced bathochromic shift associated with extended π-conjugation, whereas MPAEMA was characterized by higher-energy, blue-shifted transitions. These findings indicate that acrylate incorporation promotes low-energy electronic transitions, whereas methacrylate substitution produces a more electronically localized optical response. MEP and DOS/tDOS analyses further supported this interpretation by showing that MPAEA has a more extended electron-density distribution and a more compact distribution of frontier electronic states, whereas MPAEMA exhibits a more heterogeneous and localized electronic environment. Thermochemical analysis showed that thermal energy, heat capacity, and entropy increased systematically with temperature for all molecules, with MPAEMA exhibiting the highest values because of its larger molecular size and greater structural complexity.

NCI, DORI, and RDG analyses revealed distinct weak-interaction patterns among the studied molecules. MPAEA showed more delocalized interaction features associated with its conjugated acrylate framework, whereas MPAEMA exhibited a more heterogeneous interaction topology because of its larger and more structurally complex methacrylate framework. These results should be interpreted as comparative evidence for differences in weak-interaction topology rather than as direct quantitative measures of binding strength. Overall, the combined results demonstrate that the transformation from the acetamide framework to acrylate and methacrylate derivatives systematically modulates electronic structure, optical behavior, thermodynamic response, and weak-interaction topology. Therefore, this study provides a coherent quantum-chemical framework for understanding structure property relationships in methoxyphenyl-based functional organic systems.

## Data Availability

No datasets were generated or analysed during the current study.
